# Use of AMSR-E microwave satellite data for land surface characteristics and snow cover variation

**DOI:** 10.1016/j.dib.2016.11.006

**Published:** 2016-11-17

**Authors:** Mukesh Singh Boori, Ralph R. Ferraro, Komal Choudhary, Alexander Kupriyanov

**Affiliations:** a National Research Council (NRC), Washington DC, USA; b NOAA/NESDIS/STAR/ Satellite Climate Studies Branch and Cooperative Institute for Climate & Satellites (CICS), ESSIC, University of Maryland, College Park, MD, USA; c Scientific Research Laboratory of Automated Systems of Scientific Research (SRL-35), Samara National Research University, Samara, Russia; d Department of Geography, Bonn University, Bonn, Germany; e Image Processing Systems Institute, Samara, Russia

## Abstract

This data article contains data related to the research article entitled “Global land cover classification based on microwave polarization and gradient ratio (MPGR)” [1] and “Microwave polarization and gradient ratio (MPGR) for global land surface phenology” [2]. This data article presents land surface characteristics and snow cover variation information from sensors like EOS Advanced Microwave Scanning Radiometer (AMSR-E). This data article use the HDF Explorer, Matlab, and ArcGIS software to process the pixel latitude, longitude, snow water equivalent (SWE), digital elevation model (DEM) and Brightness Temperature (BT) information from AMSR-E satellite data to provide land surface characteristics and snow cover variation data in all-weather condition at any time. This data information is useful to discriminate different land surface cover types and snow cover variation, which is turn, will help to improve monitoring of weather, climate and natural disasters.

**Table:** 

**Specifications Table**	
Subject area	Earth and Space Science
More specific subject area	Remote Sensing, GIS and Geo-informatics
Type of data	Image, table, figure, graph
How data was acquired	Collect from Satellite Climate Studies Branch/National Oceanic and Atmospheric Administration (NOAA), and Goddard Space Flight Centre/National Aeronautics and Space Administration (NASA), and download from United States Geological Survey (USGS) website
Data format	Analyzed
Experimental factors	Image processing
Experimental features	Georeferenced, Change Detection, Image Enhancement, Band Combination, Resampling, Principal Component Analysis, Image Classification, Combined satellite data in GIS with the help of HDF Explorer, Matlab, ArcGIS software
Data source location	NOAA/NESDIS/STAR/Satellite Climate Studies Branch College Park MD, USA. Cooperative Institute for Climate and Satellites (CICS), ESSIC, University of Maryland College Park, MD, USA. Goddard Space Flight Centre NASA, Greenbelt, MD, USA
Data accessibility	Data is in this data article

**Value of the data**

• This data information is useful to understand the land surface characteristics to use in weather forecasting applications, even during cloudy and precipitation conditions which often interferes with other sensors [2], [3].
• This data information is useful for timely monitoring of natural disasters for minimizing economic losses caused by floods, drought, etc. Actually access of large-scale regional land surface information is critical to emergency management during natural disasters [4], [5].
• This data information helps us to understand how satellite remote sensing can be useful for the long-term observation of the intra and inter-annual variability of snow packs in rather inaccessible regions and providing useful information on a critical component of the hydrological cycle, where the network of meteorological stations is deficient [6], [7].
• This data information is useful for monitoring the seasonal snow cover variation for several purposes such as climatology, hydrometeorology, water use and control and hydrology, including flood forecasting and food production [8], [9].

## 1 Data

**Fig. 1:**
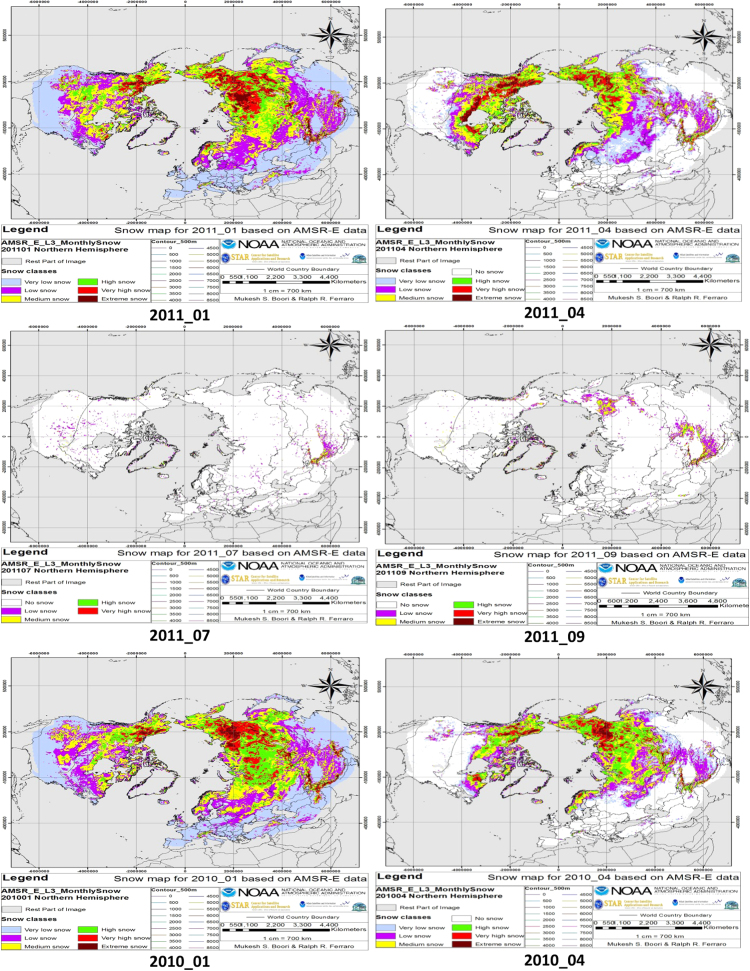

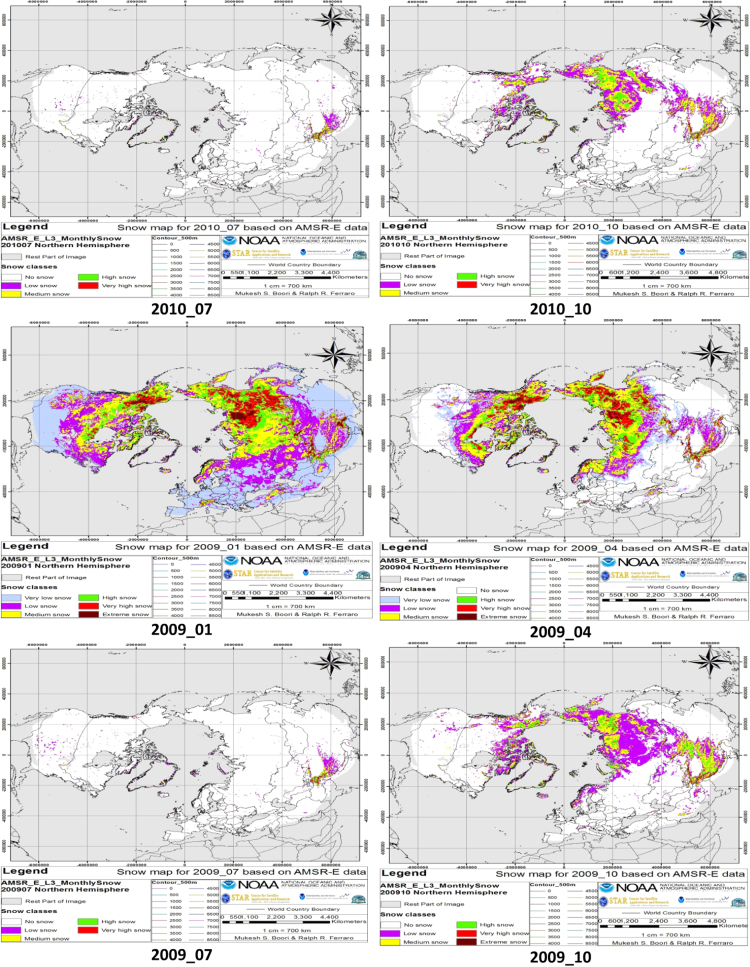

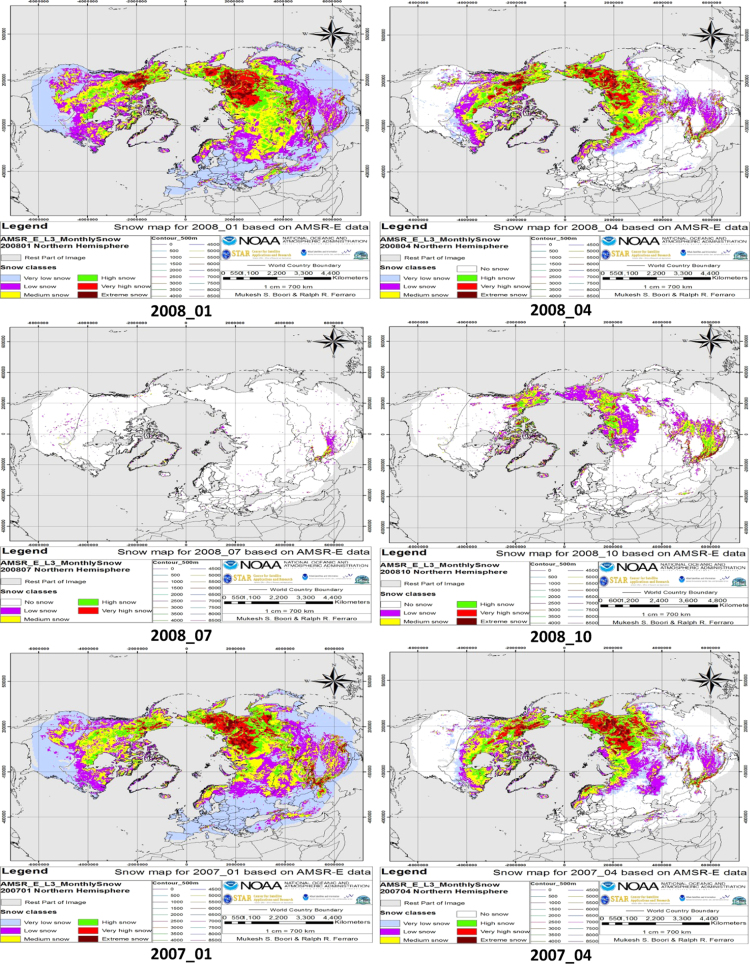

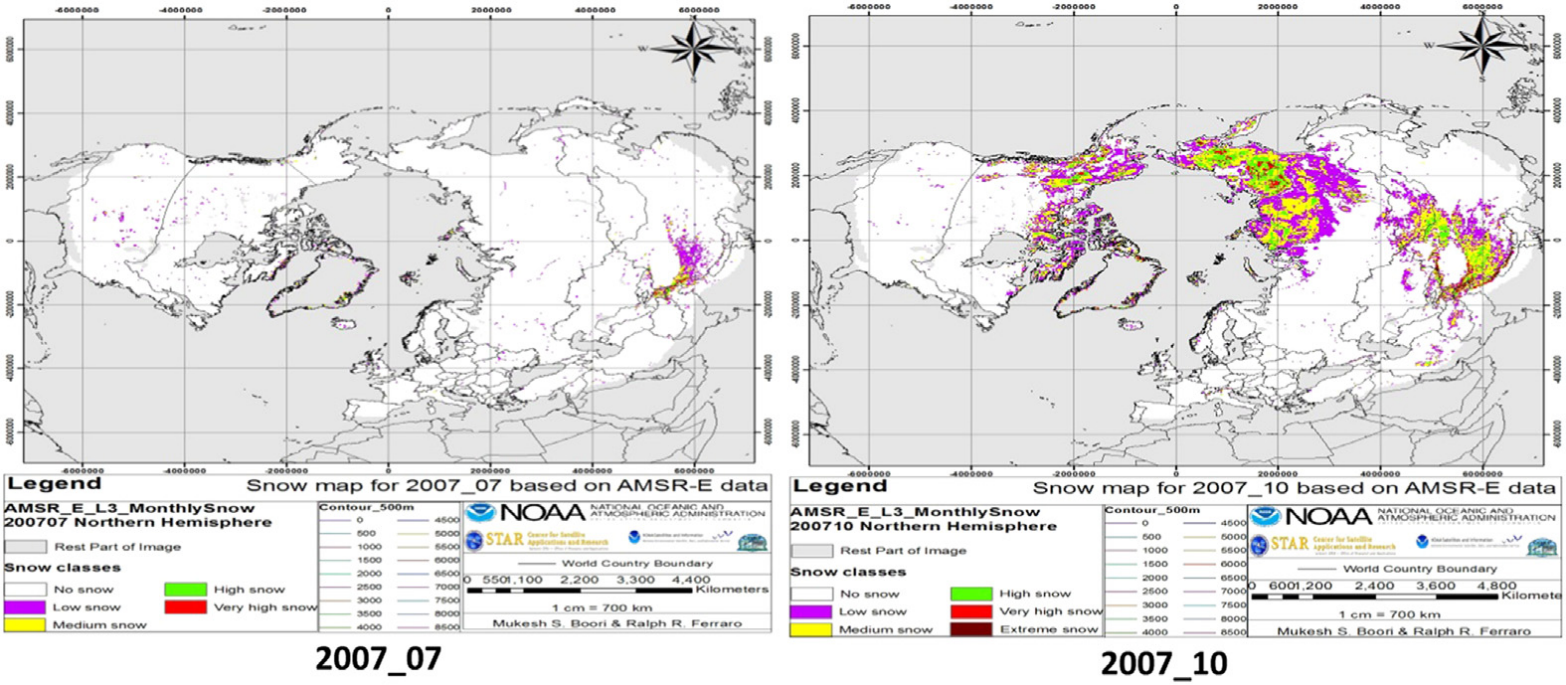
Snow cover with snow classes from 2007 to 2011 for January, April, July, and October months.

**Fig. 2:**
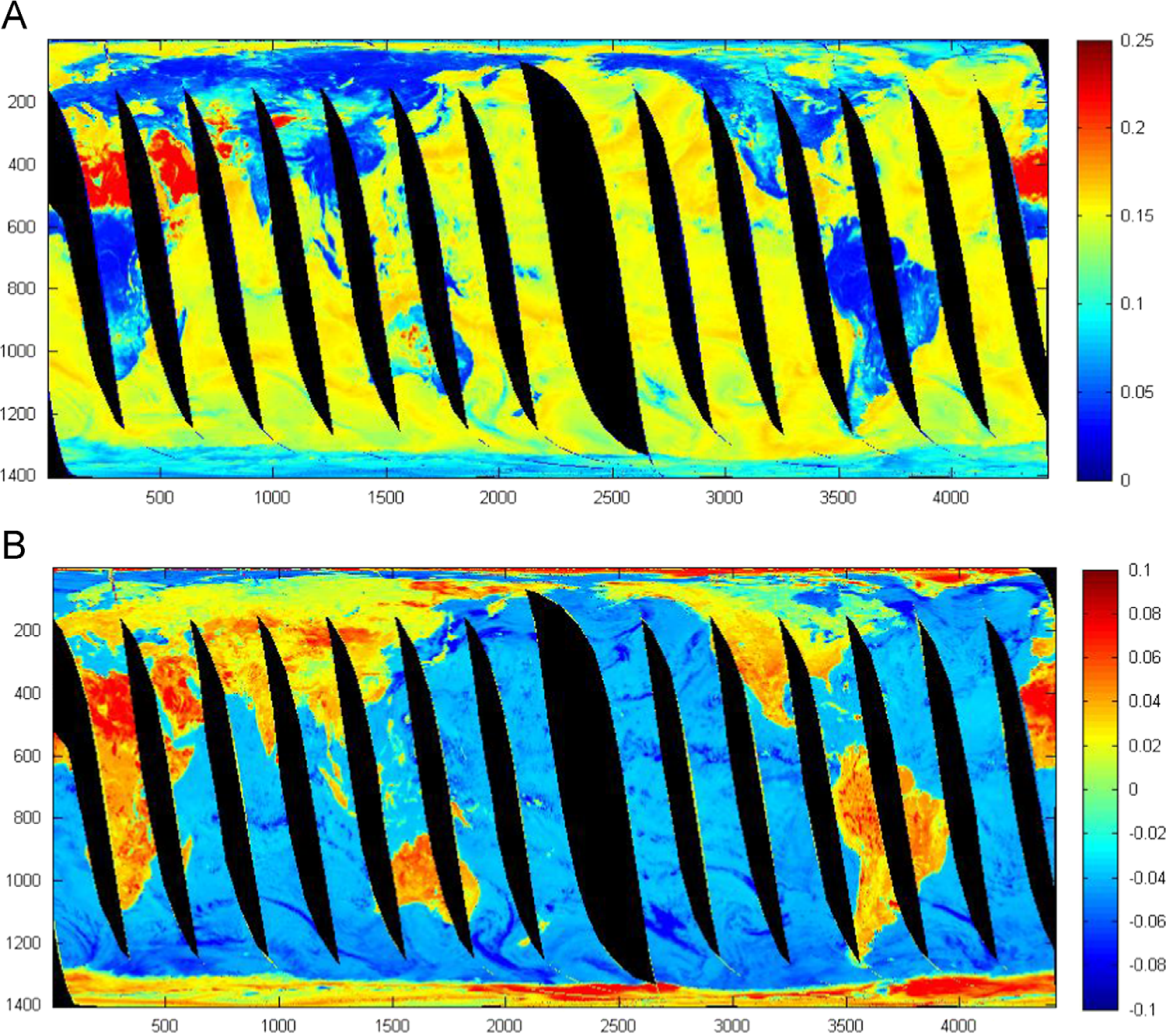
AMSR-E image with MPGR value range for (A) polarization ratio (PR 36.5) and (B) gradient ratio GR-V (36.5–18.7). In panel A, the dark red areas indicate deserts, dark blue represents dense vegetation, and the color in between correspond to mixed vegetation. In panel B, dark red highlights desert regions and light red showing vegetation condition, yellow and sky blue showing mixed vegetation (30/09/2011). Both images clearly differentiate land and water on earth after polarization or gradient ratio.

**Fig. 3:**
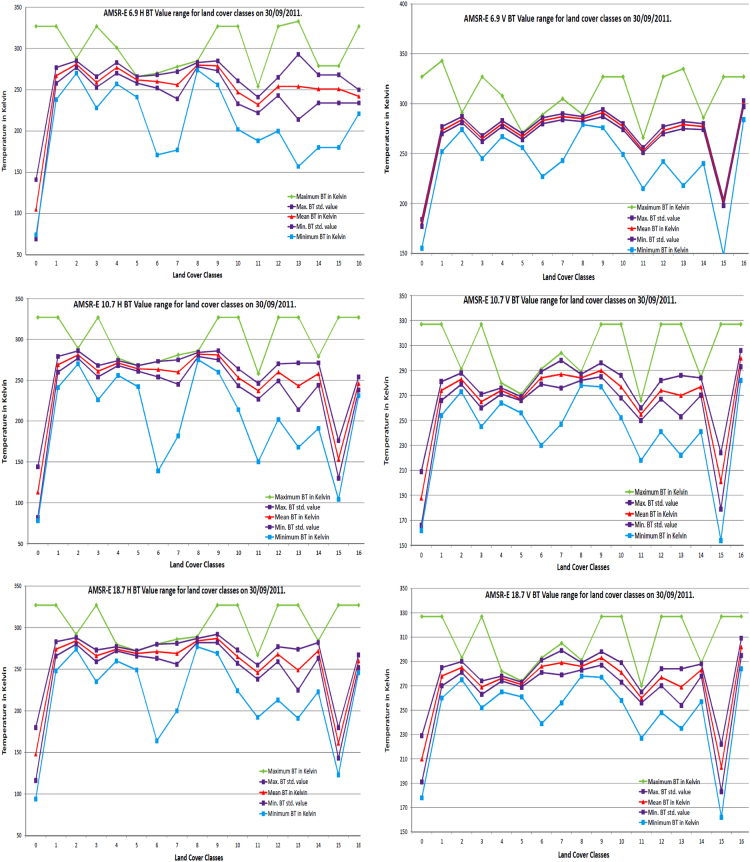

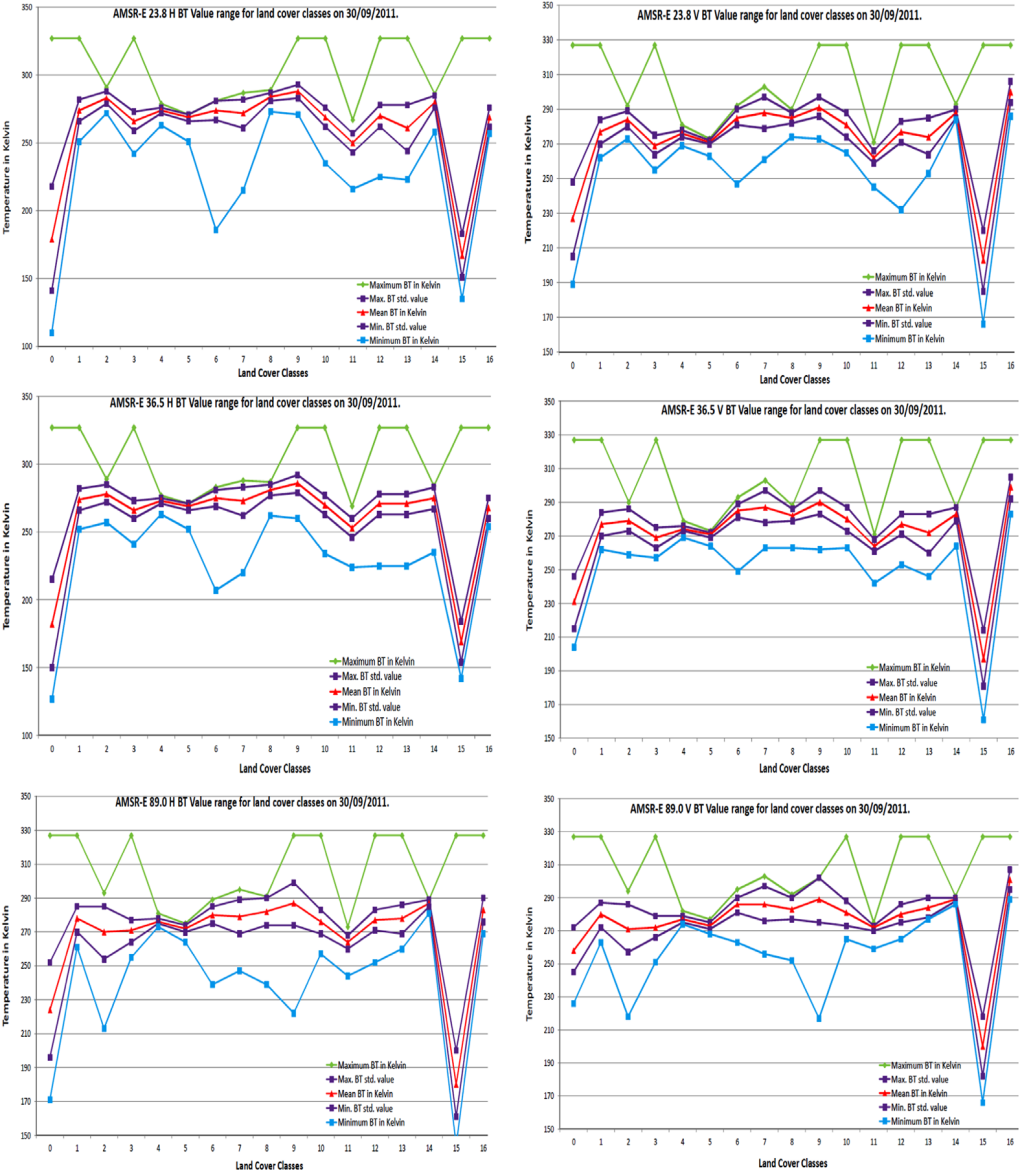
Seventeen land cover classes maximum, minimum, mean and standard deviation temperature in kelvin for 6.9, 10.7, 18.7, 23.8, 36.5 and 89.0 GHz AMSR-E frequency.

The dataset of this article provide following information:

A. Snow cover variation with seasons and elevation (Fig. 1 and Table 1, Table 2).
B. Land use/cover classified map based on MPGR values (Fig. 2 and Table 3).
C. Different frequencies actual physical land surface temperature (Fig. 3).

## 2 Experimental design, materials and methods

The experiments were carried out in Satellite Climate Studies Branch (NOAA) with the help of Goddard Space Flight Centre NASA. The Advanced Microwave Scanning Radiometer (AMSR-E) was deployed on the NASA Earth Observing System (EOS) polar-orbiting Aqua satellite platform provides global passive microwave measurements of terrestrial, oceanic and atmospheric variables for the investigation of water and energy cycles [10], [11]. The monthly level-3 AMSR-E snow water equivalent (SWE) data AE_MoSno (AMSR-E/Aqua monthly L3 Global Snow Water Equivalent EASE-Grids) in Northern Hemisphere were obtained from the NSIDC, NOAA. These data are stored in Hierarchical Data Format–Earth Observing System (HDF–EOS) format and contain SWE data and quality assurance flags mapped to 25 km Equal-Area Scalable Earth Grids (EASE-Grids). For height information Shuttle Radar Topography Mission (SRTM) data of approximately 90 m resolution were downloaded from the USGS website and used to prepare the digital elevation map (DEM). Moderate Resolution Imaging Spectroradiometer (MODIS) land cover data (MCD12Q1) was acquired from the Goddard Space Flight Centre NASA and used to determine land cover information [12], [13]. As AMSR-E satellite data was in HDF-EOS file format so first it converted into GeoTif file format with the help of HEG tool (HDF-EOS to GeoTIFF Conversion Tool, NASA) and then projected in Lambert Azimuthal equal area projection. Once data were converted into GeoTif file format, we used ArcGIS software to generate landscape and snow cover variation data.

### 2.1 Snow variation data

Snow cover classification was computed from 2007 to 2011 for the months of January, April, July and October. Separate analyses were done for every 500 m elevation ranges. The snow was classified into six main classes based on SWE values: very low snow, low snow, medium snow, high snow, very high snow and extreme snow and land which was covered by snow in winter but not in other seasons were classified as “No Snow” class. Actual SWE values are scaled down by a factor of 2 for storing in the HDF-EOS file, resulting in a stored data range of 0–240. In terms of snow depth each gray level need to multiply by factor 2. This data shows snow depth from 0 to 480mm. Fig. 1 shows the seasonal variations of the snow cover area (SCA) accumulated over the whole study area (Northern Hemisphere) for January, April, July and October months from 2007 to 2011.

Snow cover classification data maps were generated for all of the five years for January, April, July and October months shown in Fig. 1 and individual class area summarized in Table 1.

Table 2 shows a more detailed analysis of snow covered areas with every 500 m elevation difference during the 2007 to 2011 seasons, for which the dynamics of SCA was the most important.

### 2.2 Landscape data

First we selected 17 training sites for all land cover classes. Then generate their maximum, minimum, mean and standard deviation values for all horizontal and vertical AMSR-E frequencies. By this way we identify behavior of all frequencies [14]. For land cover classification we used microwave polarization and gradient ration (MPGR) combination and derive land cover data (Fig. 2).

Fig. 3 show behavior of each land cover classes for all AMSR-E data horizontal and vertical frequencies, which help to identify specify frequency for specific land cover class.

Table 3 shows all 17 land cover classes and their specific MPGR value range in a specific frequency combination.

## Figures and Tables

**Table 1:** Snow classes and snow cover area in million km^2^ for January, April, July and October months from 2007 to 2011.

**Class**	**2011_01**			**2010_01**			**2009_01**			**2008_01**			**2007_01**		
	**Area**	**%**		**Area**	**%**		**Area**	**%**		**Area**	**%**		**Area**	**%**	
Very low snow	21.9	36.4		21.4	35.7		22.2	37.0		21.7	36.2		24.3	40.6	
Low snow	13.4	22.3		13.2	21.9		15.1	25.1		14.8	24.7		13.2	22.1	
Medium snow	11.5	19.1		11.2	18.7		11.2	18.7		11.9	19.9		11.4	19.0	
High snow	7.5	12.5		8.6	14.4		6.7	11.1		6.7	11.2		6.3	10.5	
Very high snow	4.3	7.2		4.4	7.3		3.7	6.1		3.5	5.8		3.6	5.9	
Extreme snow	1.5	2.5		1.2	2.0		1.2	1.9		1.3	2.2		1.2	1.9	
**Total snow**	**60.0**	**100.0**		**60.0**	**100.0**		**60.0**	**100.0**		**60.0**	**100.0**		**60.0**	**100.0**	
RPI	264.9			264.9			264.9			264.9			264.9		
**Total**	**324.8**			**324.8**			**324.8**			**324.8**			**324.8**		
	**2011_04**			**2010_04**			**2009_04**			**2008_04**			**2007_04**		
**Class**	**Area**	**%**	**%**	**Area**	**%**	**%**	**Area**	**%**	**%**	**Area**	**%**	**%**	**Area**	**%**	**%**

Very low snow	10.7	27.8	17.8	8.6	24.2	14.3	8.8	24.3	14.7	9.5	26.6	15.9	9.6	26.7	16.0
Low snow	9.8	25.6	16.4	8.9	25.1	14.8	8.9	24.5	14.8	8.8	24.6	14.6	9.3	25.8	15.5
Medium snow	7.7	20.0	12.8	8.3	23.5	13.9	8.2	22.6	13.7	7.4	20.6	12.3	7.5	20.9	12.6
High snow	5.5	14.4	9.2	6.0	16.8	9.9	5.9	16.3	9.9	5.9	16.5	9.8	5.3	14.6	8.8
Very high snow	3.5	9.1	5.8	2.9	8.1	4.8	3.3	9.2	5.6	3.2	9.0	5.4	3.3	9.1	5.5
Extreme snow	1.1	3.0	1.9	0.8	2.2	1.3	1.1	3.1	1.9	0.9	2.6	1.6	1.1	2.9	1.8
**Total snow**	**38.4**	**100.0**	**64.0**	**35.4**	**100.0**	**59.0**	**36.2**	**100.0**	**60.5**	**35.8**	**100.0**	**59.6**	**36.1**	**100.0**	**60.1**
No snow	21.6		36.0	24.6		41.0	23.7		39.5	24.2		40.4	23.9		39.9
**Total classes**	**60.0**		**100.0**	**60.0**		**100.0**	**60.0**		**100.0**	**60.0**		**100.0**	**60.0**		**100.0**
RPI	264.9			264.9			264.9			264.9			264.9		
**Total**	**324.8**			**324.8**			**324.8**			**324.8**			**324.8**		
	**2011_07**			**2010_07**			**2009_07**			**2008_07**			**2007_07**		
**Class**	**Area**	**%**	**%**	**Area**	**%**	**%**	**Area**	**%**	**%**	**Area**	**%**	**%**	**Area**	**%**	**%**

Low snow	1.5	73.4	2.5	1.1	66.2	1.8	1.3	69.9	2.2	1.1	72.4	1.8	1.1	70.9	1.9
Medium snow	0.4	18.8	0.7	0.3	20.3	0.5	0.3	18.3	0.6	0.3	19.7	0.5	0.3	21.5	0.6
High snow	0.1	5.8	0.2	0.2	9.2	0.2	0.2	8.1	0.2	0.1	5.9	0.1	0.1	5.1	0.1
Very high snow	0.0	1.9	0.1	0.1	4.3	0.1	0.1	3.8	0.1	0.0	2.0	0.0	0.0	2.5	0.1
**Total snow**	**2.1**	**100.0**	**3.5**	**1.6**	**100.0**	**2.7**	**1.9**	**100.0**	**3.1**	**1.5**	**100.0**	**2.5**	**1.6**	**100.0**	**2.6**
No snow	57.9		96.6	58.4		97.3	58.2		96.9	58.5		97.5	58.4		97.4
**Total classes**	**60.0**		**100.0**	**60.0**		**100.0**	**60.0**		**100.0**	**60.0**		**100.0**	**60.0**		**100.0**
RPI	264.8			264.8			264.8			264.8			264.8		
**Total**	**324.8**			**324.8**			**324.8**			**324.8**			**324.8**		
	**2011_09**			**2010_10**			**2009_10**			**2008_10**			**2007_10**		
**Class**	**Area**	**%**	**%**	**Area**	**%**	**%**	**Area**	**%**	**%**	**Area**	**%**	**%**	**Area**	**%**	**%**

Low snow	2.6	59.6	4.3	7.4	54.0	12.4	11.0	62.0	18.4	7.3	54.1	12.2	7.4	52.2	12.3
Medium snow	1.2	28.4	2.1	4.4	31.7	7.3	4.4	24.8	7.4	3.5	26.2	5.9	4.4	31.1	7.3
High snow	0.4	8.1	0.6	1.7	12.5	2.9	1.9	10.9	3.2	2.0	15.0	3.4	1.8	12.6	3.0
Very high snow	0.1	2.8	0.2	0.3	1.8	0.4	0.4	2.0	0.6	0.6	4.1	0.9	0.5	3.5	0.8
Extreme snow	0.1	1.2	0.1	0.0	0.0	0.0	0.1	0.3	0.1	0.1	0.5	0.1	0.1	0.6	0.2
**Total snow**	**4.3**	**100.0**	**7.2**	**13.7**	**100.0**	**22.9**	**17.8**	**100.0**	**29.7**	**13.5**	**100.0**	**22.5**	**14.2**	**100.0**	**23.6**
No snow	55.7		92.8	46.2		77.1	42.2		70.3	46.5		77.5	45.8		76.4
**Total classes**	**60.0**		**100.0**	**60.0**		**100.0**	**60.0**		**100.0**	**60.0**		**100.0**	**59.9**		**100.0**
RPI	264.9			264.9			264.9			264.9			264.9		
**Total**	**324.8**			**324.8**			**324.8**			**324.8**			**324.8**		

**Table 2:** Snow cover area in km^2^ on 500 m elevation intervals from 0 to 8500 m for January, April, July and October months from 2007 to 2011.

**Contour**	**2011_01**		**2010_01**		**2009_01**		**2008_01**		**2007_01**	
	**Area**	**%**	**Area**	**%**	**Area**	**%**	**Area**	**%**	**Area**	**%**
0	17362649.6	32.6	17959009.5	33.0	16539754.7	30.7	17177288.2	32.7	17481910.7	32.1
500	9197864.9	17.3	10935393.3	20.1	10494707.4	19.5	9463614.5	18.0	11692291.3	21.5
1000	10294087.4	19.3	8425619.9	15.5	10313948.1	19.1	10085143.7	19.2	8252253.1	15.1
1500	4284155.9	8.0	4197795.3	7.7	4046441.8	7.5	6478086.7	12.3	4001756.6	7.3
2000	4800833.2	9.0	8012046.2	14.7	7669374.4	14.2	4443398.8	8.5	8167279.0	15.0
2500	3665846.9	6.9	1174627.0	2.2	1126771.6	2.1	1123920.2	2.1	1188233.3	2.2
3000	637913.4	1.2	628591.2	1.2	627988.2	1.2	645518.8	1.2	641266.3	1.2
3500	426450.2	0.8	400986.6	0.7	411614.3	0.8	430249.4	0.8	422342.9	0.8
4000	400835.7	0.8	405413.7	0.7	406438.4	0.8	393439.4	0.7	389942.0	0.7
4500	604524.6	1.1	580856.0	1.1	595727.4	1.1	581812.2	1.1	609286.1	1.1
5000	955138.9	1.8	951997.0	1.8	937544.2	1.7	971476.8	1.9	962679.4	1.8
5500	516529.0	1.0	524896.1	1.0	542921.1	1.0	525954.8	1.0	513200.8	0.9
6000	136987.0	0.3	138872.2	0.3	128189.7	0.2	131331.6	0.3	134473.5	0.2
6500	19479.8	0.0	17594.7	0.0	17594.7	0.0	19479.8	0.0	16966.3	0.0
7000	3141.9	0.0	3141.9	0.0	3141.9	0.0	2513.5	0.0	3141.9	0.0
7500	1256.8	0.0	1256.8	0.0	1256.8	0.0	1256.8	0.0	1256.8	0.0
8000	628.4	0.0	628.4	0.0	628.4	0.0	628.4	0.0	628.4	0.0
**Total**	**53308323.6**	**100.0**	**54358725.6**	**100.0**	**53864042.9**	**100.0**	**52475113.4**	**100.0**	**54478908.3**	100.0
	**2011_04**		**2010_04**		**2009_04**		**2008_04**		**2007_04**	
**Contour**	**Area**	**%**	**Area**	**%**	**Area**	**%**	**Area**	**%**	**Area**	**%**

0	10999024.2	30.4	7878629.6	23.5	9080069.2	26.9	7997883.2	24.8	8436200.9	26.5
500	6764994.4	18.7	13234929.7	39.4	4685703.7	13.9	7064639.2	21.9	5465795.2	17.2
1000	3436179.6	9.5	3521242.5	10.5	5145792.3	15.3	2824878.9	8.8	3415148.5	10.7
1500	8661662.3	24.0	2653021.0	7.9	7965703.4	23.6	7551817.1	23.4	2296278.4	7.2
2000	1919586.5	5.3	1469056.9	4.4	2054501.5	6.1	2015961.6	6.2	1909271.0	6.0
2500	869231.9	2.4	1361802.1	4.1	886655.9	2.6	1335973.3	4.1	6460291.5	20.3
3000	548500.3	1.5	552940.8	1.6	1021159.8	3.0	536248.4	1.7	1026730.1	3.2
3500	368967.9	1.0	355960.5	1.1	349032.5	1.0	363113.1	1.1	348273.2	1.1
4000	342367.1	0.9	319343.4	1.0	335367.3	1.0	354689.3	1.1	337351.4	1.1
4500	594704.9	1.6	573096.7	1.7	559153.8	1.7	580175.6	1.8	531475.6	1.7
5000	956298.9	2.6	993456.0	3.0	935009.0	2.8	947195.9	2.9	957132.0	3.0
5500	521782.1	1.4	508988.5	1.5	543753.1	1.6	528694.3	1.6	522184.5	1.6
6000	135730.3	0.4	136987.0	0.4	128818.1	0.4	135730.3	0.4	136358.6	0.4
6500	17594.7	0.0	18851.4	0.1	16966.3	0.1	18223.0	0.1	17594.7	0.1
7000	3141.9	0.0	3141.9	0.0	3141.9	0.0	3141.9	0.0	3770.3	0.0
7500	1256.8	0.0	1256.8	0.0	1256.8	0.0	628.4	0.0	1256.8	0.0
8000	628.4	0.0	628.4	0.0	628.4	0.0	628.4	0.0	628.4	0.0
**Total**	**36141652.0**	**100.0**	**33583333.0**	**100.0**	**33712712.8**	**100.0**	**32259621.6**	**100.0**	**31865740.9**	**100.0**
	**2011_07**		**2010_07**		**2009_07**		**2008_07**		**2007_07**	
**Contour**	**Area**	**%**	**Area**	**%**	**Area**	**%**	**Area**	**%**	**Area**	**%**

0	24977.1	5.1	16251.0	3.2	22070.7	3.6	17640.8	3.7	19238.2	3.7
500	9376.2	1.9	5903.8	1.2	4172.7	0.7	4828.9	1.0	6057.8	1.2
1000	3766.8	0.8	0.0	0.0	0.0	0.0	1486.4	0.3	0.0	0.0
1500	2717.8	0.6	1885.1	0.4	1885.1	0.3	3164.3	0.7	2513.5	0.5
2000	4927.9	1.0	3494.8	0.7	3494.8	0.6	2640.4	0.5	2238.0	0.4
2500	3374.0	0.7	3374.0	0.7	6714.0	1.1	1256.8	0.3	628.4	0.1
3000	17821.4	3.7	4172.7	0.8	20510.6	3.4	6686.2	1.4	6686.2	1.3
3500	21783.6	4.5	10230.5	2.0	21139.0	3.5	16740.3	3.5	16111.9	3.1
4000	25537.6	5.2	10230.5	2.0	24683.3	4.1	16111.9	3.4	14855.2	2.8
4500	36174.2	7.4	29109.8	5.8	34737.4	5.7	26568.4	5.5	29533.9	5.7
5000	159247.9	32.7	207542.1	41.2	230191.7	37.9	191164.9	39.8	223048.0	42.7
5500	119869.5	24.6	149102.7	29.6	181150.1	29.8	140140.4	29.2	152697.2	29.3
6000	49246.4	10.1	53054.7	10.5	46500.2	7.6	42377.8	8.8	40100.9	7.7
6500	6686.2	1.4	6283.8	1.2	6912.2	1.1	7314.6	1.5	6283.8	1.2
7000	628.4	0.1	628.4	0.1	1885.1	0.3	1256.8	0.3	628.4	0.1
7500	628.4	0.1	1285.1	0.3	1256.8	0.2	628.4	0.1	628.4	0.1
8000	628.4	0.1	628.4	0.1	628.4	0.1	628.4	0.1	628.4	0.1
**Total**	**487391.7**	**100.0**	**503177.3**	**100.0**	**607931.9**	**100.0**	**480635.6**	**100.0**	**521878.2**	**100.0**
	**2011_09**		**2010_10**		**2009_10**		**2008_10**		**2007_10**	
**Contour**	**Area**	**%**	**Area**	**%**	**Area**	**%**	**Area**	**%**	**Area**	**%**

0	111976.6	6.8	188062.3	6.4	533296.6	10.9	185548.8	5.9	218392.8	6.7
500	41247.1	2.5	47128.6	1.6	952328.4	19.4	47531.0	1.5	109753.1	3.4
1000	126556.5	7.7	197488.0	6.7	390727.4	8.0	197714.0	6.3	236673.6	7.3
1500	184694.4	11.2	417068.5	14.1	573907.7	11.7	388305.3	12.3	454947.7	14.0
2000	145332.4	8.8	304891.6	10.3	359504.6	7.3	302405.9	9.6	321080.9	9.9
2500	112882.6	6.8	205480.5	6.9	218952.8	4.5	223351.5	7.1	249665.3	7.7
3000	100544.6	6.1	149656.5	5.1	185774.7	3.8	169803.2	5.4	176804.6	5.4
3500	50474.8	3.1	105568.0	3.6	121933.7	2.5	115423.9	3.7	117507.2	3.6
4000	50877.1	3.1	86920.8	2.9	120108.8	2.5	103484.7	3.3	117683.6	3.6
4500	87929.9	5.3	214935.6	7.3	300326.2	6.1	275859.2	8.8	225362.6	6.9
5000	352463.8	21.3	610560.2	20.6	694321.3	14.2	678827.7	21.5	592111.2	18.2
5500	214404.7	13.0	342467.6	11.6	362801.7	7.4	364863.3	11.6	341210.8	10.5
6000	59696.2	3.6	78547.6	2.7	73520.6	1.5	87570.9	2.8	80432.7	2.5
6500	8169.0	0.5	8169.0	0.3	9425.7	0.2	8169.0	0.3	8797.3	0.3
7000	1885.1	0.1	1885.1	0.1	1885.1	0.0	1885.1	0.1	1885.1	0.1
7500	1256.8	0.1	1256.8	0.0	1256.8	0.0	1256.8	0.0	1256.8	0.0
8000	628.4	0.0	628.4	0.0	628.4	0.0	628.4	0.0	628.4	0.0
**Total**	**1651019.9**	**100.0**	**2960714.9**	**100.0**	**4900700.4**	**100.0**	**3152628.5**	**100.0**	**3254193.8**	**100.0**

**Table 3:** Land cover classes and there MPGR value.

**Land Cover Classes**	**PR-10**	**PR-18**	**PR-36**	**PR-89**	**GR-V (89-18)**	**GR-H (89-18)**	**GR-V (36-10)**	**GR-H (36-10)**
Water	0.20–0.25	0.17–0.18	0.035–0.04	0.06–0.07	0.10–0.11	0.20–0.25	0.10–0.11	0.30–0.4
Evergreen Needle leaf Forest	0.005–0.01	0.005–0.01	0.005–0.01	0.00–0.005	0.00–0.005	0.005–0.01	0.005–0.01	0.005–0.01
Evergreen Broad leaf Forest	0.00–0.005	0.00–0.005	0.00–0.005	0.00–0.005	−0.02 to −0.03	−0.02 to −0.03	−0.01 to −0.005	−0.01 to −0.005
Deciduous Needle leaf Forest	0.005–0.01	0.005–0.01	0.005–0.01	0.00–0.005	0.005–0.01	0.005–0.01	0.005–0.01	0.005–0.01
Deciduous Broad leaf Forest	0.005–0.01	0.00–0.005	0.00–0.005	0.00–0.005	0.00–0.005	0.00–0.005	-0.005–0.0	0.00–0.005
Mixed Forest	0.005–0.01	0.00–0.005	0.00–0.005	0.00–0.005	0.005–0.01	0.005–0.01	0.005–0.01	0.005–0.01
Closed Shrub lands	0.035–0.04	0.025–0.03	0.015–0.02	0.01–0.015	-0.005–0.0	0.015–0.02	0.00–0.005	0.02–0.025
Open Shrub lands	0.04–0.05	0.035–0.04	0.025–0.03	0.01–0.015	−0.01 to −0.005	0.015–0.02	-0.005–0.0	0.025–0.03
Woody Savannas	0.00–0.005	0.00–0.005	0.00–0.005	0.00–0.005	-0.01 to −0.005	−0.005–0.0	−0.005–0.0	−0.005–0.0
Savannas	0.015–0.02	0.01–0.015	0.005–0.01	0.00–0.005	−0.01 to −0.005	0.00–0.005	−0.005–0.0	0.005–0.01
Grasslands	0.04–0.05	0.025–0.03	0.015–0.02	0.005–0.01	-0.005–0.0	0.02–0.025	0.005–0.01	0.03–0.035
Permanent Wetlands	0.035–0.04	0.025–0.03	0.02–0.025	0.015–0.02	0.02–0.025	0.035–0.04	0.015–0.02	0.03–0.035
Croplands	0.025–0.03	0.015–0.02	0.01–0.015	0.005–0.01	0.005–0.01	0.015–0.02	0.005–0.01	0.02–0.025
Urban Built-up	0.05–0.06	0.035–0.04	0.00–0.005	0.01–0.015	0.025–0.03	0.05–0.06	0.00–0.005	0.05–0.06
Cropland Natural Vegetation Mosaic	0.03–0.035	0.02–0.025	0.01–0.015	0.00–0.005	0.01–0.015	0.025–0.03	0.01–0.015	0.03–0.035
Snow Ice	0.13–0.14	0.11–0.12	0.07–0.08	0.05–0.06	−0.01 to −0.005	0.05–0.06	−0.02 to −0.01	0.05–0.06
Barren Sparsely Vegetated	0.09–0.10	0.07–0.08	0.05–0.06	0.035–0.04	−0.005–0.0	0.04–0.05	−0.005–0.0	0.04–0.05
