# Supplementary material for: Use of AMSR-E microwave satellite data for land surface characteristics and snow cover variation
Source: Data Brief. 2016 Nov 17;9:1077–89. doi: 10.1016/j.dib.2016.11.006 (PMC5127930; doi:10.1016/j.dib.2016.11.006)
Supplement: Supplementary file 3 — Supplementary material [file mmc3.docx]

| **Land Cover Classes** | **PR-10** | **PR-18** | **PR-36** | **PR-89** | **GR-V(89-18)** | **GR-H(89-18)** | **GR-V(36-10)** | **GR-H(36-10)** |
| --- | --- | --- | --- | --- | --- | --- | --- | --- |
| Water | 0.20 – 0.25 | 0.17 – 0.18 | 0.035 – 0.04 | 0.06 – 0.07 | 0.10 – 0.11 | 0.20 – 0.25 | 0.10 – 0.11 | 0.30 – 0.4 |
| Evergreen Needle leaf Forest | 0.005 – 0.01 | 0.005 – 0.01 | 0.005 – 0.01 | 0.00 – 0.005 | 0.00 – 0.005 | 0.005 – 0.01 | 0.005 – 0.01 | 0.005 – 0.01 |
| Evergreen Broad leaf Forest | 0.00 – 0.005 | 0.00 – 0.005 | 0.00 – 0.005 | 0.00 – 0.005 | -0.02- -0.03 | -0.02- -0.03 | -0.01 - -0.005 | -0.01 - -0.005 |
| Deciduous Need leaf Forest | 0.005 – 0.01 | 0.005 – 0.01 | 0.005 – 0.01 | 0.00 – 0.005 | 0.005 – 0.01 | 0.005 – 0.01 | 0.005 – 0.01 | 0.005 – 0.01 |
| Deciduous Broad leaf Forest | 0.005 – 0.01 | 0.00 – 0.005 | 0.00 – 0.005 | 0.00 – 0.005 | 0.00 – 0.005 | 0.00 – 0.005 | -0.005 – 0.0 | 0.00 – 0.005 |
| Mixed Forest | 0.005 – 0.01 | 0.00 – 0.005 | 0.00 – 0.005 | 0.00 – 0.005 | 0.005 – 0.01 | 0.005 – 0.01 | 0.005 – 0.01 | 0.005 – 0.01 |
| Closed Shrub lands | 0.035 – 0.04 | 0.025 – 0.03 | 0.015 – 0.02 | 0.01 – 0.015 | -0.005 – 0.0 | 0.015 – 0.02 | 0.00 – 0.005 | 0.02 – 0.025 |
| Open Shrub lands | 0.04 – 0.05 | 0.035 – 0.04 | 0.025 – 0.03 | 0.01 – 0.015 | -0.01 - -0.005 | 0.015 – 0.02 | -0.005 – 0.0 | 0.025 – 0.03 |
| Woody Savannas | 0.00 – 0.005 | 0.00 – 0.005 | 0.00 – 0.005 | 0.00 – 0.005 | -0.01 - -0.005 | -0.005 – 0.0 | -0.005 – 0.0 | -0.005 – 0.0 |
| Savannas | 0.015 – 0.02 | 0.01 – 0.015 | 0.005 – 0.01 | 0.00 – 0.005 | -0.01 - -0.005 | 0.00 – 0.005 | -0.005 – 0.0 | 0.005 – 0.01 |
| Grasslands | 0.04 – 0.05 | 0.025 – 0.03 | 0.015 – 0.02 | 0.005 – 0.01 | -0.005 – 0.0 | 0.02 – 0.025 | 0.005 – 0.01 | 0.03 – 0.035 |
| Permanent Wetlands | 0.035 – 0.04 | 0.025 – 0.03 | 0.02 – 0.025 | 0.015 – 0.02 | 0.02 – 0.025 | 0.035 – 0.04 | 0.015 – 0.02 | 0.03 – 0.035 |
| Croplands | 0.025 – 0.03 | 0.015 – 0.02 | 0.01 – 0.015 | 0.005 – 0.01 | 0.005 – 0.01 | 0.015 – 0.02 | 0.005 – 0.01 | 0.02 – 0.025 |
| Urban Built-up | 0.05 – 0.06 | 0.035 – 0.04 | 0.00 – 0.005 | 0.01 – 0.015 | 0.025 – 0.03 | 0.05 – 0.06 | 0.00 – 0.005 | 0.05 – 0.06 |
| Cropland Natural Vegetation Mosaic | 0.03 – 0.035 | 0.02 – 0.025 | 0.01 – 0.015 | 0.00 – 0.005 | 0.01 – 0.015 | 0.025 – 0.03 | 0.01 – 0.015 | 0.03 – 0.035 |
| Snow Ice | 0.13 – 0.14 | 0.11 – 0.12 | 0.07 – 0.08 | 0.05 – 0.06 | -0.01 - -0.005 | 0.05 – 0.06 | -0.02 - -0.01 | 0.05 – 0.06 |
| Barren Sparsely Vegetated | 0.09 – 0.10 | 0.07 – 0.08 | 0.05 – 0.06 | 0.035 – 0.04 | -0.005 – 0.0 | 0.04 – 0.05 | -0.005 – 0.0 | 0.04 – 0.05 |

| **MPGR Range** | **PR-10** | **PR-18** | **PR-36** | **PR-89** | **GR-V**  **(89-18)** | **GR-H**  **(89-18)** | **GR-V**  **(36-10)** | **GR-H**  **(89-18)** |
| --- | --- | --- | --- | --- | --- | --- | --- | --- |
| -0.02- -0.03 |  |  |  |  | Evergreen Broad leaf Forest | Evergreen Broad leaf Forest |  |  |
| -0.02 - -0.01 |  |  |  |  |  |  | Snow Ice |  |
| -0.01 - -0.005 |  |  |  |  | Savannas, Snow Ice, Woody Savannas, Open Shrub lands |  | Evergreen Broad leaf Forest | Evergreen Broad leaf Forest |
| -0.005 – 0.00 |  |  |  |  | Barren Sparsely Vegetated/Bare Soil, Closed Shrub lands, Grasslands | Woody Savannas | Woody Savannas, Barren Sparsely Vegetated/ Bare Soil, Deciduous Broad leaf Forest, Savannas, Open Shrub lands | Woody Savannas |
| 0.00 – 0.005 | Evergreen Broad leaf Forest, Woody Savannas | Mixed Forest, Evergreen Broad leaf Forest, Woody Savannas, Deciduous Broad leaf Forest | Evergreen Broad leaf Forest, Woody Savannas, Deciduous Broad leaf Forest, Urban Built-up, Mixed Forest | Evergreen Broad leaf Forest, Woody Savannas, Deciduous Broad leaf Forest, Mixed Forest, Deciduous Need leaf Forest, Evergreen Needle leaf Forest, Savannas, Cropland Natural Vegetation Mosaic | Deciduous Broad leaf Forest, Evergreen Needle leaf Forest | Savannas, Deciduous Broad leaf Forest | Closed Shrub lands, Urban Built-up | Deciduous Broad leaf Forest |
| 0.005 – 0.01 | Deciduous Broad leaf Forest, Mixed Forest, Deciduous Need leaf Forest, Evergreen Needle leaf Forest | Deciduous Need leaf Forest, Evergreen Needle leaf Forest | Evergreen Needle leaf Forest, Deciduous Need leaf Forest, Savannas | Croplands, Grasslands | Deciduous Need leaf Forest, Croplands, Mixed Forest | Mixed Forest, Evergreen Needle leaf Forest, Deciduous Need leaf Forest | Evergreen Needle leaf Forest, Croplands, Grasslands, Deciduous Need leaf Forest, Mixed Forest | Savannas, Evergreen Needle leaf Forest, Deciduous Need leaf Forest, Mixed Forest |
| 0.01 – 0.015 |  | Savannas | Croplands, Cropland Natural Vegetation Mosaic | Closed Shrub lands, Open Shrub lands, Urban Built-up | Cropland Natural Vegetation Mosaic |  | Cropland Natural Vegetation Mosaic |  |
| 0.015 – 0.02 | Savannas | Croplands | Closed Shrub lands, Grasslands | Permanent Wetlands |  | Closed Shrub lands, Open Shrub lands, Croplands | Permanent Wetlands |  |
| 0.02 – 0.025 |  | Cropland Natural Vegetation Mosaic | Permanent Wetlands |  | Permanent Wetlands | Grasslands |  | Croplands, Closed Shrub lands |
| 0.025 – 0.03 | Croplands | Closed Shrub lands, Grasslands, Permanent Wetlands | Open Shrub lands |  | Urban Built-up | Cropland Natural Vegetation Mosaic |  | Open Shrub lands |
| 0.03 – 0.035 | Cropland Natural Vegetation Mosaic |  |  |  |  |  |  | Grasslands, Cropland Natural Vegetation Mosaic, Permanent Wetlands |
| 0.035 – 0.04 | Permanent Wetlands, Closed Shrub lands | Open Shrub lands, Urban Built-up | Water | Barren Sparsely Vegetated/ Bare Soil |  | Permanent Wetlands |  |  |
| 0.04 – 0.05 | Grasslands, Open Shrub lands |  |  |  |  | Barren Sparsely Vegetated |  | Barren Sparsely Vegetated |
| 0.05 – 0.06 | Urban Built-up |  | Barren Sparsely Vegetated/Bare Soil | Snow Ice |  | Snow Ice, Urban Built-up |  | Snow Ice, Urban Built-up |
| 0.06 – 0.07 |  |  |  | Water |  |  |  |  |
| 0.07 – 0.08 |  | Barren Sparsely Vegetated | Snow Ice |  |  |  |  |  |
| 0.08 – 0.09 |  |  |  |  |  |  |  |  |
| 0.09 – 0.10 | Barren Sparsely Vegetated |  |  |  |  |  |  |  |
| 0.10 – 0.11 |  |  |  |  | Water |  | Water |  |
| 0.11 – 0.12 |  | Snow Ice |  |  |  |  |  |  |
| 0.12 – 0.13 |  |  |  |  |  |  |  |  |
| 0.13 – 0.14 | Snow Ice |  |  |  |  |  |  |  |
| 0.14 – 0.15 |  |  |  |  |  |  |  |  |
| 0.15 – 0.16 |  |  |  |  |  |  |  |  |
| 0.17 – 0.18 |  | Water |  |  |  |  |  |  |
| 0.18 – 0.19 |  |  |  |  |  |  |  |  |
| 0.19 – 0.20 |  |  |  |  |  |  |  |  |
| 0.20 – 0.25 | Water |  |  |  |  | Water |  |  |
| 0.25 – 0.30 |  |  |  |  |  |  |  |  |
| 0.30 – 0.40 |  |  |  |  |  |  |  | Water |
